# Hepatocyte-Specific RORα Deficiency Accelerates Liver Regeneration by Unleashing ME1-Driven Glycolysis

**DOI:** 10.3390/genes17091029

**Published:** 2026-08-28

**Authors:** Hongmei Zhang, Shanshan Yang, Ming Yi, Qiuyue Guan, Dan Ding, Xiaoqian Yu, Yin Liu, Dan Deng, Yuxi Feng, Zhiguang Su

**Affiliations:** 1 Institute of High Altitude Medicine, Center for High Altitude Medicine, High Altitude Medicine Key Laboratory of Sichuan Province, West China Hospital, Sichuan University, Chengdu 610041, China; 2 Frontiers Science Center for Disease-Related Molecular Network, West China Hospital, Sichuan University, Chengdu 610041, China; 3 Department of Outpatient, Sichuan Provincial People’s Hospital, University of Electronic Science and Technology of China, Chengdu 610041, China; 4 Department of Pain Management, West China Hospital, Sichuan University, Chengdu 610041, China; 5 Department of Laboratory Medicine, West China Hospital, Sichuan University, Chengdu 610041, China; 6 West China Biobank, West China Hospital, Sichuan University, Chengdu 610041, China; 7 College of Physics, Sichuan University, Chengdu 610041, China

**Keywords:** liver regeneration, hepatocyte proliferation, RORα, glycolysis, malic enzyme 1

## Abstract

**Background/Objectives**: Hepatocyte proliferation during liver regeneration requires precise transcriptional coordination of metabolic reprogramming, but the key regulators governing this process remain largely unknown. **Methods:** Hepatocyte-specific retinoic acid receptor-related orphan receptor α (RORα) knockout (RORα-LKO) mice were generated and subjected to two-thirds partial hepatectomy (PHx). Liver regeneration was assessed by liver-to-body weight ratio, histology, and proliferation markers (PCNA, Ki67, cyclin D1). Metabolic changes were evaluated by untargeted metabolomics, Seahorse extracellular flux analysis, and glycolytic enzyme activity assays. The molecular mechanism was investigated through RNA-seq analysis, qPCR, Western blotting, dual-luciferase reporter assays, CUT&Tag-qPCR, and pharmacological inhibition. **Results:** RORα expression was transiently upregulated during the early phase and declined at the peak of hepatocyte proliferation. RORα-LKO mice exhibited accelerated liver recovery and increased hepatocyte proliferation following a partial hepatectomy, without enhanced inflammation or fatty acid oxidation. Mechanistically, RORα directly binds to the promoter of *Me1* to repress its transcription. Loss of RORα upregulates ME1, enhances glycolysis, and increases ATP production. Pharmacological inhibition of ME1 reversed these effects. **Conclusions:** The RORα–ME1 axis links transcriptional repression to metabolic reprogramming and may represent a therapeutic target for liver regeneration.

## 1. Introduction

The liver possesses a remarkable capacity to regenerate following surgical resection, toxic injury, or viral insult. Upon loss of hepatic mass, the remaining quiescent hepatocytes rapidly re-enter the cell cycle in a highly synchronized manner to restore liver mass and function within days. This regenerative process is orchestrated by a complex interplay of cytokine signaling, growth factor cascades, cell-cycle machinery, and extensive tissue remodeling [1]. Recent evidence has established that metabolic reprogramming is not merely a consequence of proliferation but an essential driver of successful liver regeneration [2]. During the early regenerative phase, hepatocytes undergo a transient metabolic shift characterized by enhanced glycolysis, altered lipid handling, and accelerated nucleotide synthesis to meet the substantial energy and biosynthetic demands of rapid cell division [3]. These coordinated metabolic adaptations are governed at the transcriptional level by specific transcription factors that sense extracellular cues and intracellular nutrient status [4]. However, the specific regulators orchestrating this process during liver regeneration remain poorly understood.

Retinoic acid receptor-related orphan receptor α (RORα) is a constitutively active member of the nuclear receptor superfamily, which also includes RORβ and RORγ. While RORβ is predominantly expressed in neural and sensory tissues and RORγ plays distinct roles in immune regulation and metabolism [5], RORα stands out for its broad involvement in circadian rhythm, lipid and glucose homeostasis, inflammatory modulation, and tumorigenesis [6,7,8]. These distinctive and pleiotropic functions make RORα a uniquely relevant candidate for linking transcriptional regulation to metabolic adaptation during hepatocyte proliferation. In various malignancies, RORα expression is frequently downregulated, and its restoration suppresses tumor cell proliferation by restraining cell-cycle progression and glycolytic metabolism [9]. Notably, RORα has been shown to inhibit glycolytic flux in hepatocellular carcinoma by attenuating key glycolytic enzymes and limiting Warburg-like metabolic reprogramming [10]. Mechanistically, RORα functions as a transcriptional regulator that directly binds to specific response elements in target gene promoters, thereby modulating metabolic gene networks.

It is critical to recognize that regenerative proliferation of normal hepatocytes differs substantially from neoplastic proliferation in terms of regulatory mechanisms, proliferative kinetics, and differentiation status [11,12]. Tumor cell proliferation is sustained by dysregulated oncogenic signaling and loss of normal growth-control mechanisms, whereas liver regeneration is a tightly regulated and self-limited physiological response that restores tissue mass and function. Thus, the role of RORα in normal regenerative biology cannot be extrapolated directly from its tumor-suppressive activities. Despite its established functions in metabolism and tumor suppression, the physiological role of RORα in liver regeneration has remained largely unexplored.

In this study, we employed hepatocyte-specific RORα knockout (RORα-LKO) mice in a two-thirds partial hepatectomy model and combined untargeted metabolomics to investigate the function and mechanism of RORα in liver regeneration. We demonstrate that RORα deficiency accelerates hepatocyte proliferation and liver mass restoration by relieving transcriptional repression of malic enzyme 1 (ME1), thereby enhancing glycolytic flux and ATP production. These findings establish the RORα–ME1 axis as a critical regulator of metabolic reprogramming during liver regeneration. From a translational perspective, delineating this regulatory mechanism may inform strategies to promote hepatic recovery after extensive resection or transplantation, where regenerative capacity is often rate-limiting for clinical outcomes.

## 2. Material and Methods

### 2.1. Mice and Treatment

The study was conducted in accordance with the Declaration of Helsinki and the animal study protocol was approved by the local Ethics Committee for Animal Research at Sichuan University. Mice were housed under a 12-h light–dark cycle with free access to food and water.

C57BL/6J *Rorα^loxp/loxp^* mice with LoxP sites flanking exon 3 of the *Rorα* gene were generated by Biocytogen Pharmaceuticals Co., Ltd. (Beijing, China). Alb-Cre mice, which express Cre recombinase specifically in hepatocytes under the control of the albumin promoter, were purchased from The Jackson Laboratory (Bar Harbor, ME, USA). To generate hepatocyte-specific *Rorα* knockout (*Rorα*-LKO) mice with a *Rorα^loxp/loxp^*; *Cre* genotype, *Rorα^loxp/loxp^* animals were crossbred with Alb-Cre mice and subsequently backcrossed. Age- and sex-matched littermates with a *RORα^loxp/loxp^* genotype were used as controls.

For two-thirds partial hepatectomy (PHx), male mice aged 8–10 weeks were anesthetized with 1% pentobarbital sodium. A midline laparotomy was performed, followed by aseptic removal of the central and left lobes. Mice in the sham control group underwent midventral laparotomy without liver manipulation [13]. Animals were euthanized at various time points after surgery. The regenerated liver was weighed, and the liver-body weight ratio was calculated. Liver tissues were collected for subsequent molecular and biochemical analyses.

### 2.2. Histology, Immunohistochemistry, and Immunostaining

As described previously [14], paraffin-embedded liver sections were stained with hematoxylin and eosin (H&E) and examined under a light microscope (Olympus, Tokyo, Japan). Lipid accumulation in hepatocytes was assessed by Oil Red O (HY-D1168, MedChemExpress, Monmouth Junction, NJ, USA). For immunohistochemistry and immunostaining, sections were incubated with monoclonal antibodies against PCNA (1:400 dilution, HuaBio, Hangzhou, China) or Ki67 (1:400 dilution, HuaBio, China) according to standard procedures. Images were captured using an Axio Imager A2 fluorescence microscope (Carl Zeiss, Oberkochen, Germany).

### 2.3. Cell Culture, Cell Proliferation and Cell Viability

Mouse AML12 cells (ATCC, USA) were selected as the principal in vitro model. These non-tumorigenic hepatocyte-derived cells are of murine origin, making them compatible with the murine hepatocyte-specific RORα knockout model used in vivo, and they provide a relevant system for investigating hepatocyte proliferation and metabolic regulation outside the context of constitutive oncogenic signaling [14]. AML12 cells were cultured in DMEM supplemented with 10% fetal bovine serum (FBS) at 37 °C in a humidified atmosphere containing 5% CO_2_.

For the EdU proliferation assay, cells were seeded approximately 1 × 10^4^ cells/well in 24-well plates. Following the indicated treatments, cells were incubated with EdU solution (C0071S, Beyotime Biotechnology, Shanghai, China) for 2 h, and EdU incorporation was measured according to the manufacturer’s instructions. Cell viability was assessed using a CCK-8 kit (EK-5103, ECOTOP, Guangzhou, China) following the manufacturer’s protocol. Briefly, cells were seeded approximately 7.5 × 10^3^ cells /well in 96-well plates. After treatment, cells were washed twice with PBS and incubated with 100 μL phenol-red-free medium containing 10% CCK-8 solution at 37 °C for 1–3 h. Absorbance was measured at 450 nm using an automatic microplate reader (Synergy H1, Bio-tek, Winooski, VT, USA).

### 2.4. RORα Overexpression, RORα Knockdown, and Dual-Luciferase Reporter Assay

The cDNAs encoding mouse *Rorα* were amplified using gene-specific primers (Supplementary Table S1) and subcloned into the pLVX-puro vector to generate the RORα overexpression construct. For RORα knockdown, small interfering RNA (siRNA) (5′-CCAGACATTGTGCGACTTCAT-3′) targeting *Rorα* was purchased from Vigene Biosciences (Shandong, China). Both constructs were transfected into AML12 cells as indicated.

To generate reporter constructs, a series of the *Me1* promoter region was PCR-amplified with specific primers and cloned into the pGL3-basic luciferase reporter vector. The putative RORα response element (RORE, AGGTCA) within the *Me1* promoter was mutated using a site-directed mutagenesis kit with the primers containing specific mutations (Supplementary Table S1). Dual-luciferase reporter assays were performed as described previously [15]. Briefly, HEK293 cells were co-transfected with the *Me1* promoter construct (wild-type or mutant) or control vector, together with RORα expression plasmid or empty vector. Luciferase activities were measured using the Dual-Glo luciferase system.

### 2.5. Targeted Cut&Tag-qPCR Assay

Targeted Cut&Tag-qPCR was performed utilizing the Hieff NGSG-Type In-Situ DNA Binding Profiling Library Prep kit for Illumina (12598ES48, Yeasen, Shanghai, China) according to the manufacturer’s instructions. Briefly, approximately 50,000 AML12 cells were immobilized on pre-washed Concanavalin A-coated magnetic beads for 10 min and then incubated with anti-RORα antibody (Abcam, ab256799) overnight at 4 °C. Normal rabbit IgG was included in parallel as a negative control. Following secondary antibody incubation, samples were incubated with pA/G-Tnp transposome, and tagmented DNA was subsequently purified. The recovered DNA was amplified by PCR to generate CUT&Tag libraries, which were then used directly for targeted qPCR analysis with specific primers (Supplementary Table S2) to amplify a 206-bp region containing the predicted RORE within the *Me1* promoter (GRCm39, NC_000075.7, chr9:86579060–8657926).

For normalization, a DNA spike-in was used as an exogenous control, and a separate primer pair (Supplementary Table S2) targeting the spike-in DNA sequence was included. The Ct value of the *Me1* promoter region was normalized to the corresponding spike-in Ct value to obtain ΔCt. Relative enrichment was calculated using the 2^−ΔΔCt^ method, with the IgG sample used as the calibrator (set to 1). Results are expressed as fold enrichment relative to IgG. Three independent biological replicates were performed for each condition.

### 2.6. LC-MS/MS-Based Metabolomic Profiling

Metabolic profiling of liver specimens was conducted using liquid chromatography–tandem mass spectrometry (LC-MS/MS) as described earlier [16]. In brief, ~10 mg of liver tissue was rinsed with cold PBS and homogenized in 1 mL of a precooled mixture (methanol/acetonitrile/water, 2:2:1, *v*/*v*/*v*). Following vortexing, the samples were sonicated in an ice-water bath for 10 min and then cleared by centrifugation (12,000× *g*) at 4 °C for 15 min. The supernatant was transferred to an AB SCIEX Triple Quad 5500 LC-MS/MS platform for metabolite detection. To minimize potential analytical drift, samples were injected in a randomized injection sequence. The retention time and peak area of each metabolite were recorded for identification and quantification. Each group contained at least three biological replicates.

Raw mass spectrometry data were processed using Analyst software (version 1.6.3) and MultiQuant software (version 3.0.2) for peak extraction to obtain metabolite-related information, including mass-to-charge ratio (*m/z*), retention time, and peak area. Subsequent data preprocessing and statistical analyses were performed using R software (version 3.4.0). For quality control, ions with missing values in more than 50% of QC samples or experimental samples were excluded, and unstable ions with a relative standard deviation (RSD) >20% across QC samples were removed. Remaining missing values were imputed using the k-nearest neighbors (KNN) algorithm, followed by median normalization. Principal component analysis (PCA) was performed on the normalized data to assess overall sample distribution and analytical stability.

Differential metabolites between groups were identified using a combination of univariate and multivariate statistical analyses. Student’s *t*-test and principal component analysis (PCA) were performed, and metabolites satisfying *p* < 0.1 and variable importance in projection (VIP) > 1 were considered differential metabolites. Hierarchical clustering analysis was used to visualize differences in metabolite abundance between groups. Pathway annotation of differential metabolites was performed using the Kyoto Encyclopedia of Genes and Genomes (KEGG) Human database, and pathway enrichment was evaluated using a hypergeometric test, with all identified metabolites used as the background set.

### 2.7. Metabolic Analysis

As previously described [15,17], plasma, hepatic and cellular levels of triglyceride, total cholesterol, ATP, alanine transaminase (ALT), and aspartate transaminase (AST), as well as the activities of phosphofructokinase (PFK), pyruvate kinase (PK), and hexokinase (HK), were measured using commercially available assay kits (Supplementary Table S3) according to the manufacturers’ instructions.

### 2.8. Extracellular Acidification Rate (ECAR) and Oxygen Consumption Rate (OCR) Measurements

ECAR reflects net extracellular proton production, which includes contributions from both glycolysis and mitochondrial respiration, and serves as an indirect measure of glycolytic flux under the experimental conditions used. The OCR directly reflects mitochondrial respiration. Real-time changes in ECAR and OCR were measured using an XF24 extracellular flux analyzer (Seahorse Bioscience, Lexington, MA, USA). AML12 cells were seeded in standard XF24 microplates at a density of 100,000 cells per well and cultured overnight at 37 °C in a humidified incubator containing 5% CO_2_. The sensor cartridge was hydrated overnight at 37 °C in a non-CO_2_ incubator and calibrated with Seahorse XF Calibrant according to the manufacturer’s instructions. On the day of the assay, the culture medium was replaced with the corresponding pre-warmed Seahorse XF assay medium, and the cells were incubated at 37 °C in a non-CO_2_ incubator for approximately 1 h before measurement.

For the mitochondrial stress test, the assay medium was supplemented with 10 mM glucose, 2 mM glutamine, and 1 mM pyruvate. After baseline OCR measurements, oligomycin (1.5 μM), FCCP (2 μM), and rotenone/antimycin A (Rot/AA; 0.5 μM) were sequentially injected. OCR was continuously monitored before and after each injection. Basal respiration was calculated as baseline OCR minus non-mitochondrial respiration. ATP-linked respiration was determined from the decrease in OCR following oligomycin treatment. Maximal respiration was calculated from the FCCP-stimulated OCR after subtraction of non-mitochondrial respiration, and spare respiratory capacity was calculated as maximal respiration minus basal respiration. The residual OCR following Rot/AA treatment was considered non-mitochondrial respiration.

For the glycolysis stress test, cells were incubated in Seahorse XF assay medium without glucose or pyruvate. After baseline ECAR measurements, glucose (10 mM), oligomycin (1 μM), and 2-deoxy-D-glucose (2-DG; 50 mM) were sequentially injected. ECAR was continuously monitored before and after each injection. Non-glycolytic acidification was defined as the ECAR measured before glucose addition. Glycolysis was calculated as the increase in ECAR following glucose addition relative to the pre-glucose baseline. Glycolytic capacity was determined from the maximum ECAR following oligomycin treatment after correction for non-glycolytic acidification, and glycolytic reserve was calculated as glycolytic capacity minus glycolysis. The reduction in ECAR following 2-DG treatment was used to confirm the glycolytic contribution to extracellular acidification.

At the end of each assay, cellular protein content in each well was determined using the Bradford method, and OCR and ECAR values were normalized to total protein content. Three independent experiments were performed.

### 2.9. RNA Extraction, cDNA Synthesis, and Real-Time Quantitative PCR (qPCR)

Total RNA was extracted from mouse liver tissues or AML12 cells using the RNeasy Mini Kit (Vazyme Biotech, Nanjing, China) as described previously [17]. cDNA synthesis and subsequent qPCR were performed using the HiScript III First Strand cDNA Synthesis Kit and AceQ qPCR SYBR Green Master Mix (Vazyme Biotech), respectively, with gene-specific primers (Supplementary Table S2) following the manufacturer’s protocols. All qPCR assays were carried out with an annealing temperature of 60 °C. Melting-curve analysis was performed at the end of each run to confirm amplification specificity. Relative mRNA levels were normalized to 18S rRNA using the comparative Ct (ΔΔCt) method.

### 2.10. Western Blotting Analysis

Protein extraction from mouse liver tissues or AML12 cells and Western blotting were performed as described previously [18]. Total protein concentration was determined using the bicinchoninic acid (BCA) assay. Antibodies against β-actin (1:5000, M1210-2), ME1 (ER63901), PCNA (1:1000, EM111201) and cyclin D1 (1:1000, ER0722) were purchased from HuaBio (Hangzhou, China). The antibody against RORα (1:1000, ab256799) was obtained from Abcam (Cambridge, UK). Primary antibodies were detected using horseradish peroxidase (HRP)-conjugated secondary antibodies, and protein signals were visualized with enhanced chemiluminescence reagents (Thermo Fisher Scientific, Waltham, MA, USA). Band intensities were quantified by densitometric analysis using ImageJ software (version 2.14.0/1.54f, National Institutes of Health, Bethesda, MD, USA) and normalized to β-actin levels in the corresponding samples.

### 2.11. RORA Expression in Human Liver Tissues

Hepatic RORA expression data were retrieved from The Cancer Genome Atlas (TCGA) and Genotype-Tissue Expression (GTEx) datasets, and analyzed using the Gene Expression Profiling Interactive Analysis (GEPIA) platform to compare hepatocellular carcinoma with normal liver tissues. Expression values were presented on a log2(TPM + 1) scale, with |log_2_ fold change| ≥ 1 and *p*-value < 0.01 considered statistically significant.

### 2.12. Statistical Analysis

Statistical analyses were performed using GraphPad Prism (version, 11.0.2, GraphPad Software Inc., La Jolla, CA, USA). Comparisons between two independent groups were performed using an unpaired two-tailed Student’s *t*-test. For experiments involving two independent variables, such as genotype and time after PHx, two-way ANOVA followed by Sidak’s multiple-comparisons test was used. For comparisons among more than two groups involving a single independent variable, one-way ANOVA followed by Tukey’s multiple-comparisons test was used. The specific statistical test used for each experiment is indicated in the corresponding figure legend. Data were presented as the mean ± standard deviation (SD). A two-side *p* value < 0.05 was considered statistically significant.

## 3. Results

### 3.1. Time-Dependent Expression Changes in RORα Following Partial Hepatectomy (PHx)

**Figure 1:**
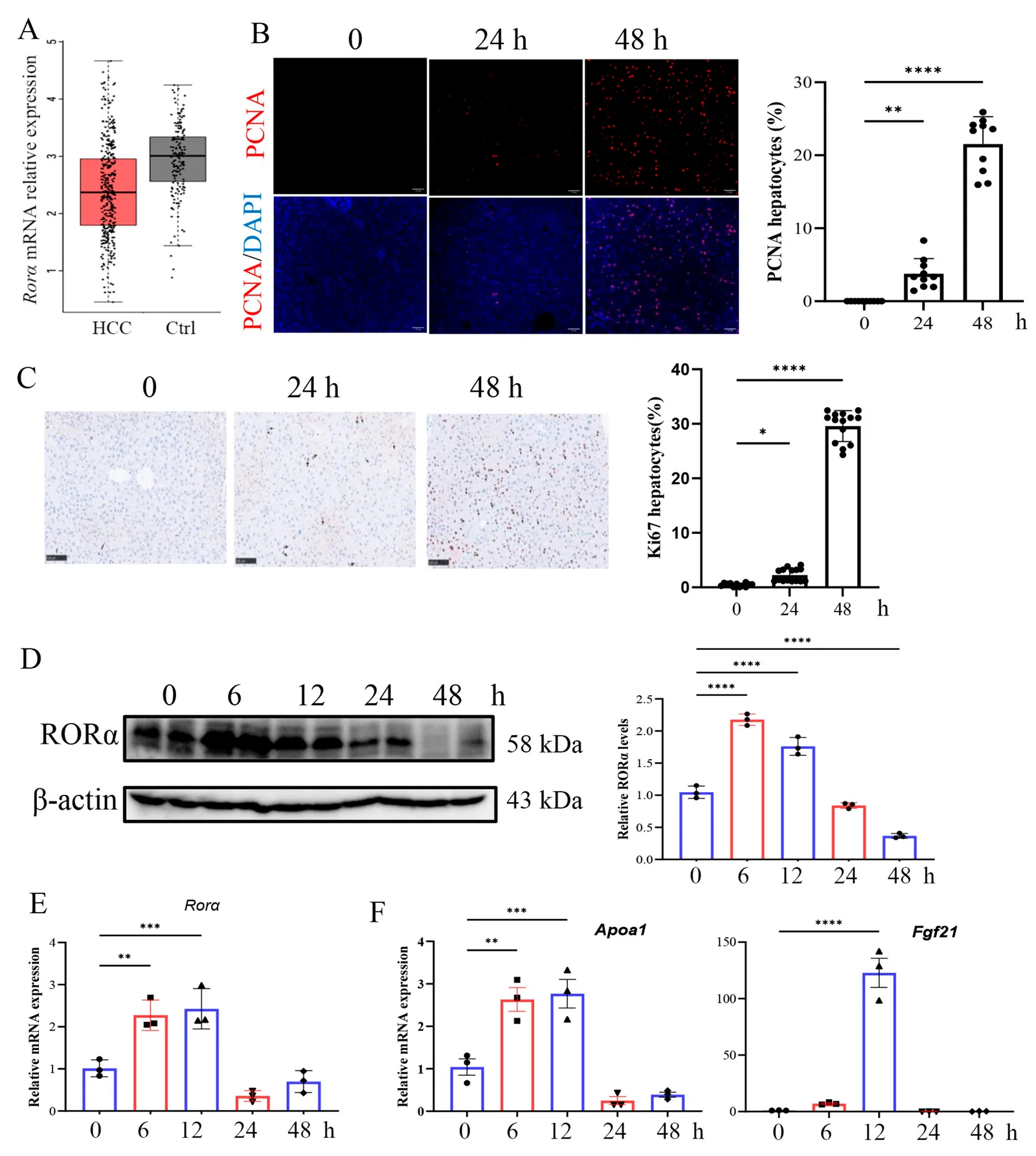
**Time-dependent changes in RORα expression during liver regeneration following partial hepatectomy (PHx).** (**A**) *Rorα* mRNA expression in hepatocellular carcinoma (HCC) versus normal control liver tissues analyzed using the GEPIA platform with TCGA and GTEx data. Red (tumor tissues, n = 374) and gray (normal tissues, n = 160) boxes show the interquartile range (IQR) and median, dots indicate individual samples. (**B**–**F**) Male mice aged 8–10 weeks were subjected to two-thirds PHx and analyzed at the indicated time points after surgery. (**B**) Representative immunofluorescence staining of PCNA (red) in liver sections, with DAPI (blue) counterstaining of nuclei, and quantification of PCNA-positive hepatocytes at 0, 24, and 48 h after PHx. (**C**) Representative immunohistochemical staining of Ki67 and quantification of Ki67-positive hepatocytes at 0, 24, and 48 h after PHx. (**D**) Western blot analysis and densitometric quantification of hepatic RORα protein expression at 0, 6, 12, 24, and 48 h after PHx. β-Actin was used as the loading control. (**E**) Hepatic *Rorα* and (**F**) its target genes *Apoa1* and *Fgf21* mRNA expression at the indicated time points after PHx were examined. Scale bar, 50 μm (**B**,**C**). Data are presented as mean ± SD (n = 3–4 per group). Statistical significance among multiple time points was assessed using one-way ANOVA followed by Tukey’s multiple-comparisons test. * *p* < 0.05, ** *p* < 0.01, *** *p* < 0.001, and **** *p* < 0.0001; specific comparisons are indicated by horizontal lines.

RORα has been shown to suppress cell proliferation in multiple tumor types [9,19,20]. Consistently, RORα expression was significantly reduced in hepatocellular carcinoma (HCC) tissues compared with normal liver tissues (Figure 1A), suggesting that RORα loss may contribute to dysregulated hepatocyte proliferation. However, neoplastic proliferation differs substantially from regenerative proliferation of normal hepatocytes in terms of regulatory mechanisms, proliferative rate, and differentiation status [11]. To investigate the expression dynamics of RORα during physiologic hepatocyte proliferation, we employed the two-thirds PHx model, a well-established in vivo system for studying liver regeneration. In this model, hepatocyte proliferation is known to peak during the 24–48-h period after PHx. Accordingly, we assessed the proliferation markers PCNA and Ki67 at 24 h and 48 h. As expected, PCNA expression (Figure 1B) and the proportion of Ki67-positive hepatocytes (Figure 1C) began to rise at 24 h after PHx and were most prominent at 48 h. In contrast, the early 6- and 12-h time points were included primarily to characterize the dynamic changes in RORα expression during the priming phase. RORα protein abundance showed a marked increase at 6 h after PHx and subsequently declined progressively to its lowest level at 48 h (Figure 1D). RORα mRNA was markedly elevated at 6 h, peaked at 12 h, and returned to low levels by 48 h (Figure 1E). The mRNA expression of RORα target genes, fibroblast growth factor 21 (*FGF21*) and apolipoprotein A1 (*APOA1*), followed an identical pattern, peaking at 12 h and declining at 24 h and 48 h (Figure 1F). These results demonstrate that RORα is transiently activated during the early phase after PHx and declines upon peak hepatocyte proliferation, implying a negative regulatory role for RORα in hepatocyte proliferation and liver regeneration.

### 3.2. Hepatocyte-Specific RORα Deficiency Accelerates Hepatocyte Proliferation and Liver Regeneration

**Figure 2:**
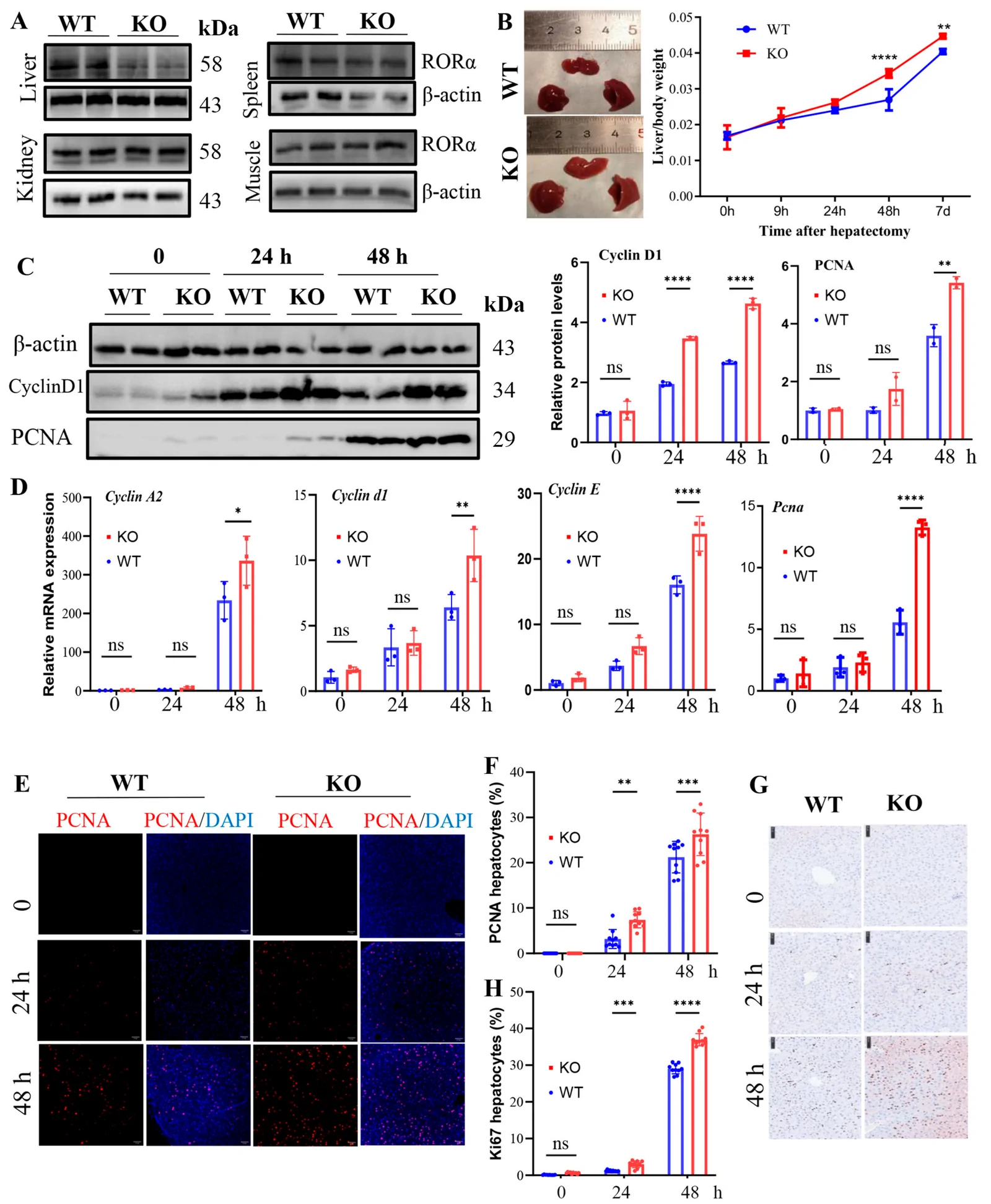
**Hepatocyte-specific RORα deficiency accelerates liver regeneration and hepatocyte proliferation.** (**A**) Western blotting validation of RORα knockout efficiency in various tissues from *Rorα* wild-type (WT) and *Rorα*-LKO (KO) mice. (**B**–**F**) *Rorα* WT and *Rorα*-LKO mice were subjected to two-thirds partial hepatectomy (PHx). Liver-to-body weight ratio (**B**), protein levels of cyclin D1 and PCNA (**C**), mRNA expression of cyclin A2, cyclin D1, cyclin E, and PCNA (**D**), PCNA-positive hepatocytes (**E**,**F**), and Ki67-positive hepatocytes (**G**,**H**) were examined at the indicated time points after PHx. Scale bar: 50 μm (**E**,**G**). Band intensities in WB were quantified by densitometric analysis using ImageJ software ( version 2.14.0/1.54f ) and normalized to β-actin levels in the corresponding samples. Data are presented as mean ± SD (n = 4–5 per group). * *p* < 0.05, ** *p* < 0.01, *** *p* < 0.001, **** *p* < 0.0001 vs. *Rorα* WT; ns, not significant.

To investigate the role of RORα in liver regeneration, we generated hepatocyte-specific RORα knockout (RORα-LKO) mice and subjected them, together with littermate RORα-fl/fl controls, to two-thirds PHx. RORα expression was efficiently depleted in the liver but remained unchanged in other tissues (Figure 2A). Baseline liver-to-body weight ratios were comparable between the two genotypes. At 9 h after PHx, the ratios remained similar between groups; thereafter, however, RORα-LKO mice exhibited accelerated liver mass restoration, as evidenced by a larger remnant liver and a significantly elevated liver-to-body weight ratio at 48 h (Figure 2B). By day 7 after PHx, although the ratio remained significantly different between the two groups, the gap was narrowing (Figure 2B), indicating that the effect of RORα deficiency on liver mass recovery is most prominent during the early-to-mid phase and attenuates over time.

We next examined whether the accelerated liver mass recovery was attributable to enhanced hepatocyte proliferation. Consistent with the rapid restoration of liver mass recovery, protein levels of cyclin D1 and PCNA were markedly elevated in RORα-LKO livers at 24 h and remained higher at 48 h (Figure 2C). Transcript levels of cell cycle regulators, including cyclin A2, cyclin D1, cyclin E, and PCNA, were also consistently upregulated in RORα-LKO mice at various time points after PHx (Figure 2D). Immunofluorescence for PCNA (Figure 2E) and immunohistochemistry for Ki-67 (Figure 2F) further confirmed a marked expansion of the proliferating hepatocyte pool in RORα-LKO mice at both 24 h and 48 h. These results demonstrate that hepatocyte-specific RORα ablation promotes hepatocyte proliferation and accelerates liver regeneration.

### 3.3. RORα Deficiency Attenuates the Priming Phase of Liver Regeneration

Liver regeneration proceeds through three sequential phases, including priming, proliferation, and termination phases [21]. During the early priming phase, inflammatory cytokines and growth factors are released to drive quiescent hepatocytes into the cell cycle. Given the enhanced proliferative capacity of RORα-LKO hepatocytes, we hypothesized that RORα deficiency might accelerate regeneration by potentiating the early priming response. To capture the acute inflammatory and growth factor signals that define the priming phase, we measured serum TNF-α and IL-6 levels and hepatic mRNA expression of transforming growth factor α (TGF-α) and hepatocyte growth factor (HGF) during the early postoperative period (up to 6 h after PHx). Because these cytokines typically surge within hours after PHx and then decline rapidly, earlier time points (3 and 6 h) were selected to specifically assess priming-associated responses. By contrast, liver injury markers ALT and AST were monitored over a broader time window (up to 48 h) to evaluate the temporal pattern of hepatocellular injury and recovery, as these enzymes are released more gradually and persist longer in the circulation.

**Figure 3:**
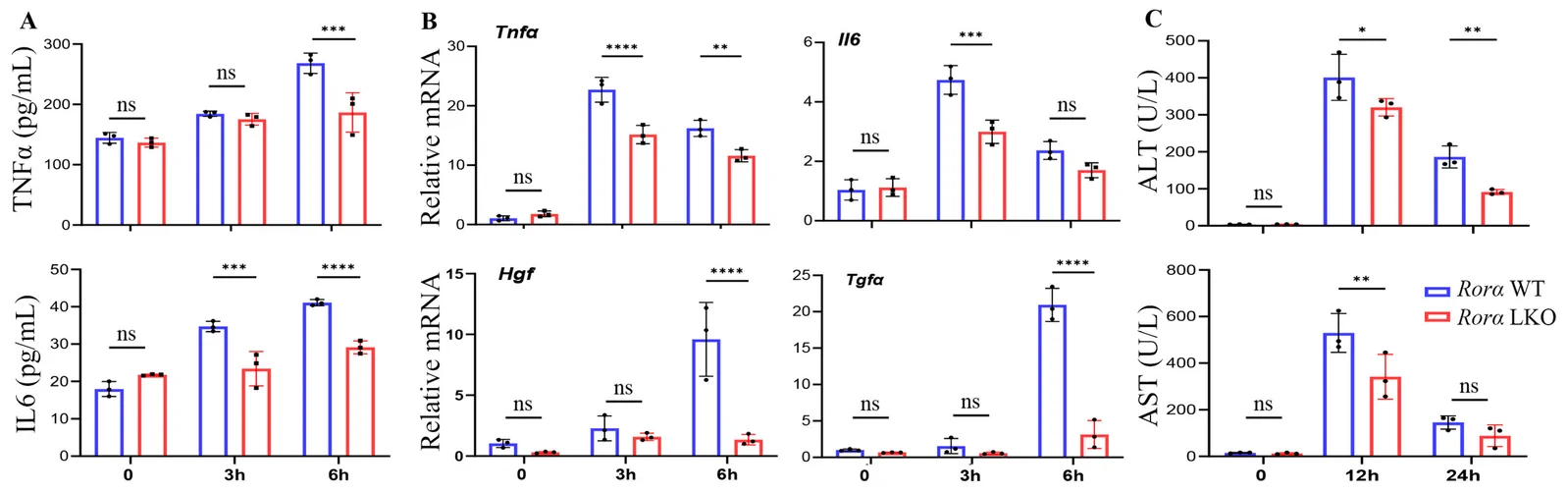
**RORα deficiency does not enhance the priming phase of liver regeneration.** *Rorα* WT and *Rorα*-LKO mice were subjected to two-thirds partial hepatectomy (PHx). Serum levels of TNF-α and IL-6 (**A**), hepatic *Tnfα*, *Il6*, *Tgfα*, and *Hgf* mRNA expression (**B**), and serum ALT and AST levels (**C**) were determined at indicated time points after surgery. Data are presented as mean ± SD (n = 3–5 per group). * *p* < 0.05, ** *p* < 0.01, *** *p* < 0.001, **** *p* < 0.0001 vs. *Rorα* WT; ns, not significant.

Before PHx, serum TNF-α and IL-6 levels were comparable between the two groups (Figure 3A). In the early phase after PHx, both cytokines increased rapidly in both groups, confirming effective induction of the priming response. However, these increases were more robust in RORα WT mice than RORα-LKO mice (Figure 3A). Accordingly, hepatic mRNA expression of *Tnfα*, *Il6*, and growth factors *Tgfα* and *Hgf* at 3 h and 6 h after PHx was even lower in RORα-LKO mice than in controls (Figure 3B). The ALT and AST levels rose rapidly after PHx in both groups (Figure 3C). Notably, RORα-LKO mice exhibited significantly lower ALT and AST levels than controls after PHx (Figure 3C), indicating that RORα deficiency attenuates rather than exacerbates hepatocellular injury.

Together, these findings demonstrate that the accelerated regeneration in RORα-LKO mice is not mediated by an enhanced priming response; rather, RORα-deficiency attenuates the inflammatory and growth factor signals. This paradox—accelerated regeneration despite reduced priming—suggests that the pro-regenerative effect of RORα loss operates through a cell-autonomous mechanism rather than through amplification of the classic priming cascade.

### 3.4. The Pro-Regenerative Effect of RORα Deficiency Is Not Associated with Enhanced Fatty Acid Metabolism

Hepatocyte proliferation during regeneration demands substantial energy and biosynthetic precursors, necessitating extensive metabolic reprogramming [22]. During early liver regeneration, hepatocytes transiently accumulate substantial amounts of lipids, a phenomenon known as transient regeneration-associated steatosis (TRAS), which may provide energy for cell proliferation [23,24]. We therefore examined whether altered lipid metabolism contributes to the accelerated regeneration observed in RORα-deficient mice.

**Figure 4:**
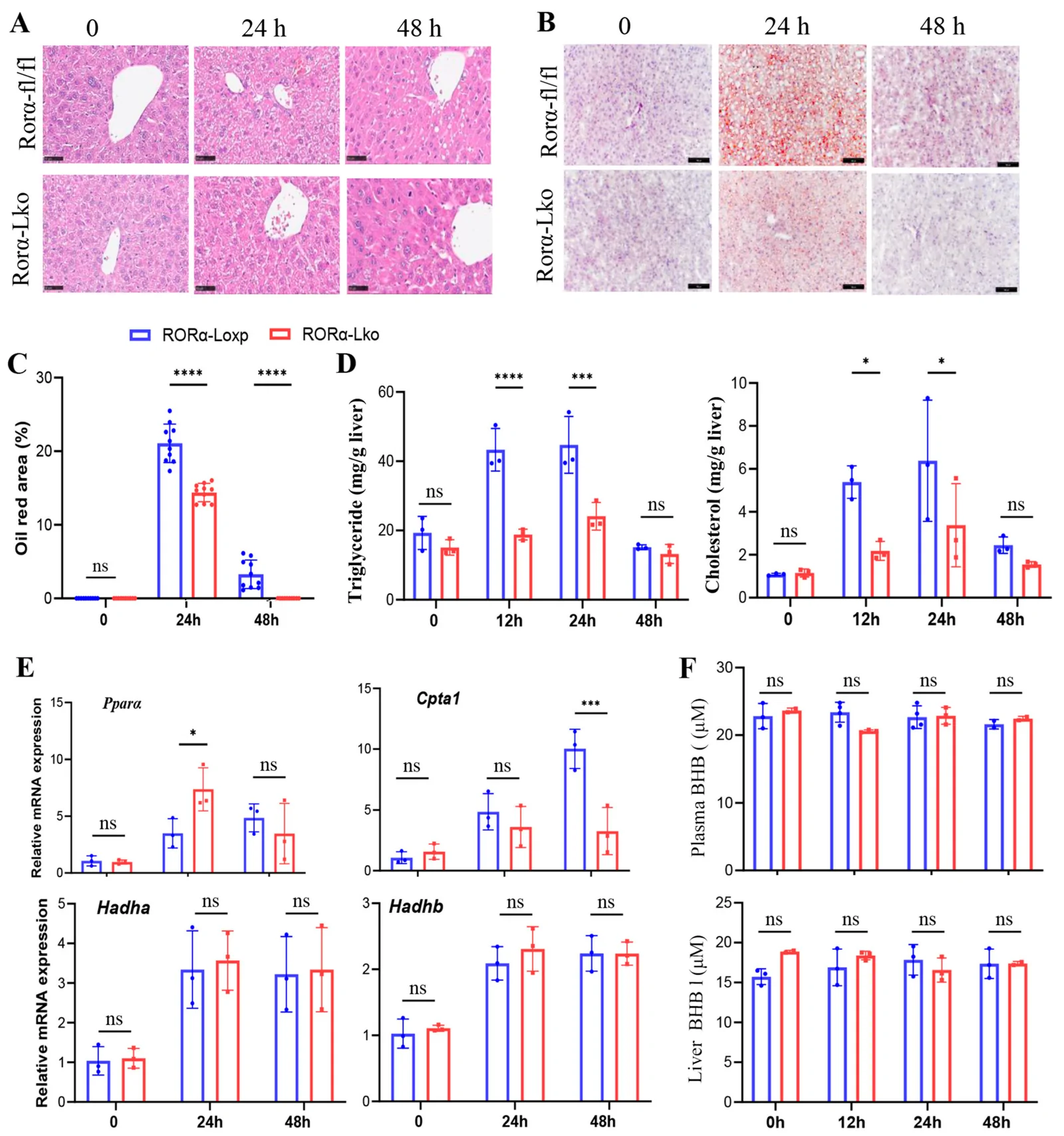
**The pro-regenerative effect of RORα deficiency is not associated with enhanced fatty acid metabolism.** *Rorα* WT and *Rorα*-LKO mice were subjected to two-thirds partial hepatectomy (PHx). At the indicated time points after surgery, (**A**) representative H&E staining of liver sections (scale bar, 50 μm); (**B**,**C**) representative Oil Red O staining of liver sections (scale bar: 50 μm) (**B**) and the quantification of Oil Red O-positive areas (**C**); (**D**) hepatic triglyceride and total cholesterol levels, (**E**) mRNA expression of fatty acid oxidation-related genes (*Ppara*, *Cpt1a*, *Hadha*, and *Hadhb*), and (**F**) serum β-hydroxybutyrate (BHB) levels. Data are presented as mean ± SD (n = 3 per group). * *p* < 0.05, *** *p* < 0.001, **** *p* < 0.0001 vs. *Rorα* WT; ns, not significant.

Histological analysis and Oil Red O staining revealed prominent lipid deposition in control livers at 24 h after PHx, whereas lipid accumulation was markedly reduced in RORα-LKO livers (Figure 4A–C). Quantification of hepatic triglycerides and total cholesterol showed no baseline differences between genotypes. Following PHx, both parameters increased in both groups, but the increases were significantly blunted in RORα-LKO mice (Figure 4D). Previous studies have demonstrated that following PHx, the regenerated liver relies heavily on fatty acid β-oxidation for energy production [25]. To explore whether reduced lipid accumulation might reflect enhanced fatty acid oxidation, we examined the expression of genes involved in this pathway, including peroxisome proliferator-activated receptor alpha (*Ppara*), carnitine palmitoyltransferase 1A (*Cpt1a*), and the hydroxyacyl-CoA dehydrogenase trifunctional multienzyme complex subunits alpha and beta (*Hadha* and *Hadhb*). Expression of these genes did not differ consistently between the two genotypes after PHx (Figure 4E). Moreover, either serum or liver β-hydroxybutyrate (BHB) levels, a hallmark metabolite of fatty acid oxidation, was not elevated in RORα-LKO mice (Figure 4F). Collectively, these findings indicate that the pro-regenerative effect of RORα deficiency is not driven by enhanced fatty acid oxidation. However, the observed reduction in hepatic lipid accumulation suggests that RORα deficiency alters lipid metabolism; whether this reduction actively contributes to accelerated regeneration remains to be determined.

### 3.5. RORα Deficiency Is Associated with Enhanced Glycolytic Features During Liver Regeneration

**Figure 5:**
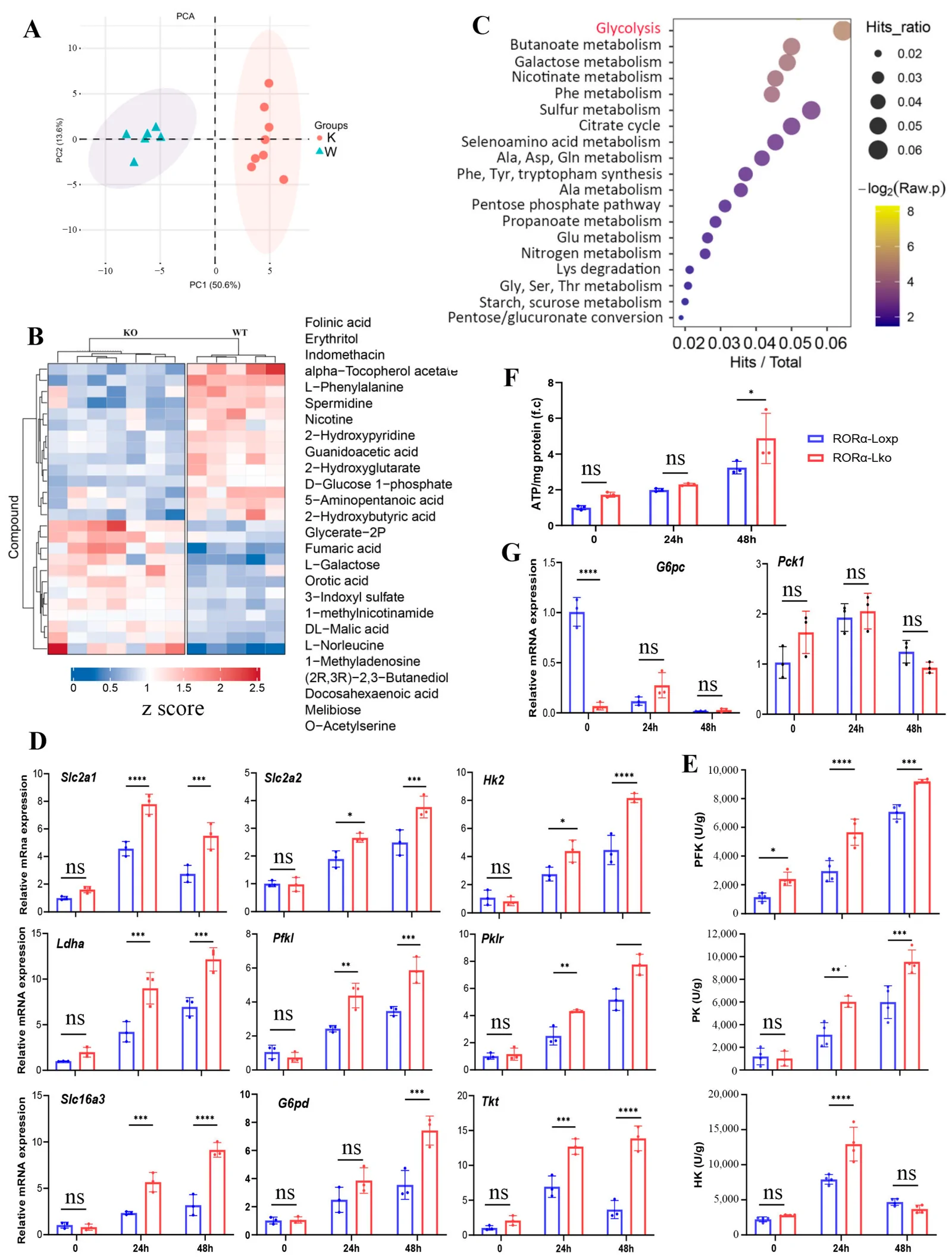
**RORα deficiency promotes glycolysis during liver regeneration.** *Rorα* WT and *Rorα* LKO mice were subjected to two-thirds PHx. (**A**–**C**) Hepatic metabolomic analysis was performed at 24 h after PHx: (**A**) principal component analysis (PCA) score plot of metabolomic profiles, (**B**) heatmap showing differentially abundant glycolysis-related metabolites (colors represent Z-score-normalized metabolite abundance), and (**C**) KEGG pathway enrichment analysis of differentially abundant metabolites. (**D**–**F**) At the indicated time points after PHx, mRNA expression of glycolytic and pentose phosphate pathway genes (*Slc2a1*, *Slc2a2*, *Hk2*, *Ldha*, *Pfkl*, *Pklr*, *Slc16a3*, *G6pd*, and *Tkt*) (**D**), activities of PFK, PK, and HK in liver lysates (**E**), hepatic ATP levels (**F**), and mRNA expression of gluconeogenic genes *G6pc* and *Pck1* (**G**) were examined. Data are presented as mean ± SD (n = 3 per group). * *p* < 0.05, ** *p* < 0.01, *** *p* < 0.01, **** *p* < 0.0001 vs. *Rorα-fl/fl*.

To obtain an unbiased assessment of how RORα deficiency affects hepatic metabolism, we performed untargeted metabolomics on livers from RORα WT and RORα-LKO mice following PHx. Principal component analysis (PCA)) revealed a clear separation of metabolic profiles between the two genotypes (Figure 5A), indicative of substantial metabolic reprogramming induced by RORα loss. Among the differentially abundant metabolites, those involved in glycolysis, including pyruvate, glucose-6-phosphate, 2-phospho-D-glycerate, and glucose-1-phosphate, exhibited the most prominent alterations (Figure 5B). Consistently, KEGG analysis identified glycolysis as the top significantly enriched pathway (Figure 5C).

We next examined mRNA expression of genes involved in glucose uptake and glycolysis, including the glucose transporters Glut1 (*Slc2a1*) and Glut2 (*Slc2a2*), hexokinase 2 (*Hk2*), lactate dehydrogenase A (*Ldha*), liver-type phosphofructokinase (*Pfkl*), pyruvate kinase L/R (*Pklr*), the lactate exporter *Mct4* (*Slc16a3*), and *G6pd* (oxidative branch) and *Tkt* (non-oxidative branch) of the pentose phosphate pathway. Under basal conditions, no significant differences were observed between the two groups. Following PHx, all these genes were upregulated in both groups, but the induction was consistently stronger in RORα-LKO mice (Figure 5D). Accordingly, activities of key glycolytic enzymes, including PFK, PK, and HK, were also significantly elevated in RORα-LKO livers (Figure 5E). Moreover, ATP levels were higher in RORα-LKO mice at 48 h after PHx (Figure 5F). In contrast, expression of the gluconeogenic genes *G6pc* and *Pck1* was suppressed at 48 h post-PHx (Figure 5G). suggesting a metabolic shift from glucose production toward glucose utilization, a hallmark of proliferating cells that rely on glycolysis for rapid ATP generation and biosynthetic precursors. Notably, G6pc expression also differed between the two genotypes under basal conditions, with lower levels observed in RORα-LKO livers (Figure 5G), suggesting that RORα deficiency may influence hepatic glucose metabolism even before regenerative stress. Collectively, these findings indicate that RORα deficiency is associated with a metabolic profile characterized by enhanced glycolytic features during liver regeneration, with evidence of baseline differences in gluconeogenic gene expression that may predispose RORα-deficient hepatocytes to a more glycolytic phenotype upon regenerative challenge.

### 3.6. Enhanced Glycolysis Is Required for RORα Deficiency-Promoted Hepatocyte Proliferation

**Figure 6:**
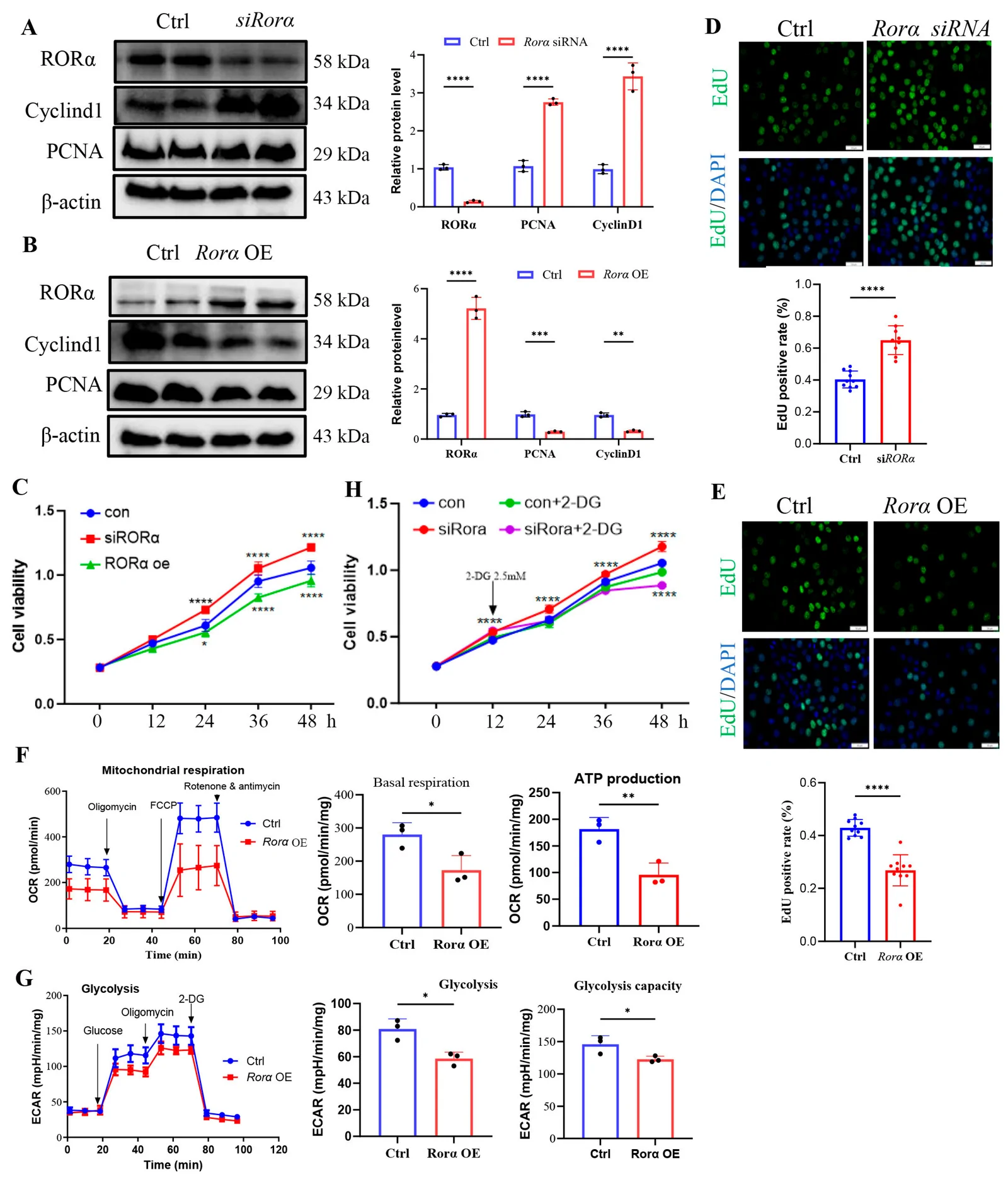
**Enhanced glycolysis is required for RORα deficiency-driven hepatocyte proliferation in AML12 cells.** (**A**) Western blot validation of RORα knockdown (siRORα) in AML12 cells. (**B**) Western blot validation of RORα overexpression (RORα-OE) and corresponding PCNA and cyclin D1 levels. (**C**) CCK-8 assay of cell viability in siRORα and RORα-OE cells. (**D**,**E**) EdU incorporation assay showing DNA synthesis in siRORα (**D**) and RORα-OE (**E**) cells. Scale bar, 50 μm. (**F**,**G**) Seahorse metabolic analysis showing ECAR (**F**) and OCR (**G**) in RORα-OE versus control cells, with quantification of basal glycolysis, glycolytic capacity, basal respiration, and ATP production. (**H**) CCK-8 assay of siRORα cells treated with or without 2-DG (2.5 mM). Data are presented as mean ± SD (n = 3 independent experiments). Statistical significance was determined using two-tailed Student’s *t*-test for comparisons between two groups, or one-way ANOVA followed by Tukey’s post hoc test for comparisons involving multiple groups. * *p* < 0.05, ** *p* < 0.01, *** *p* < 0.001, **** *p* < 0.0001 vs. control.

Given the confounding effects of inflammation and hemodynamic alterations inherent to the in vivo environment, we determined whether RORα directly regulates hepatocyte proliferation and glycolysis in the AML12 hepatocyte cell line. RORα was knocked down or overexpressed using siRNA or an expression plasmid, respectively (Figure 6A,B). CCK-8 assays revealed that RORα knockdown significantly increased cell viability, whereas its overexpression reduced it (Figure 6C). In agreement, EdU incorporation was elevated upon RORα knockdown but diminished upon RORα overexpression (Figure 6D,E). Western blotting further confirmed that RORα knockdown upregulated PCNA and cyclin D1, whereas RORα overexpression downregulated these proliferation-associated proteins (Figure 6A,B). These findings establish that RORα suppresses DNA synthesis and cell proliferation.

We subsequently measured the extracellular acidification rate (ECAR) and oxygen consumption rate (OCR) to assess glycolytic flux and mitochondrial oxidative phosphorylation, respectively [26]. RORα-overexpressing cells exhibited reduced basal glycolysis, glycolytic capacity, basal respiration, and ATP production (Figure 6F,G), indicating that RORα suppresses both glycolysis and energy metabolism. To test whether enhanced glycolysis is necessary for the proliferative advantage conferred by RORα-knockdown, we treated siRORα cells with 2-deoxy-D-glucose (2-DG), a glucose analog that competitively inhibits glucose utilization and thereby blocks glycolysis [27]. Notably, 2-DG treatment markedly attenuated the increased viability of siRORα cells (Figure 6H).

Collectively, these results demonstrate that RORα suppresses both glycolysis and proliferation in hepatocytes, and that enhanced glycolysis is indispensable for the pro-proliferative effect of RORα deficiency.

### 3.7. RORα Inhibits Hepatic Glycolysis and Proliferation Through Transcriptional Suppression of Me1

**Figure 7:**
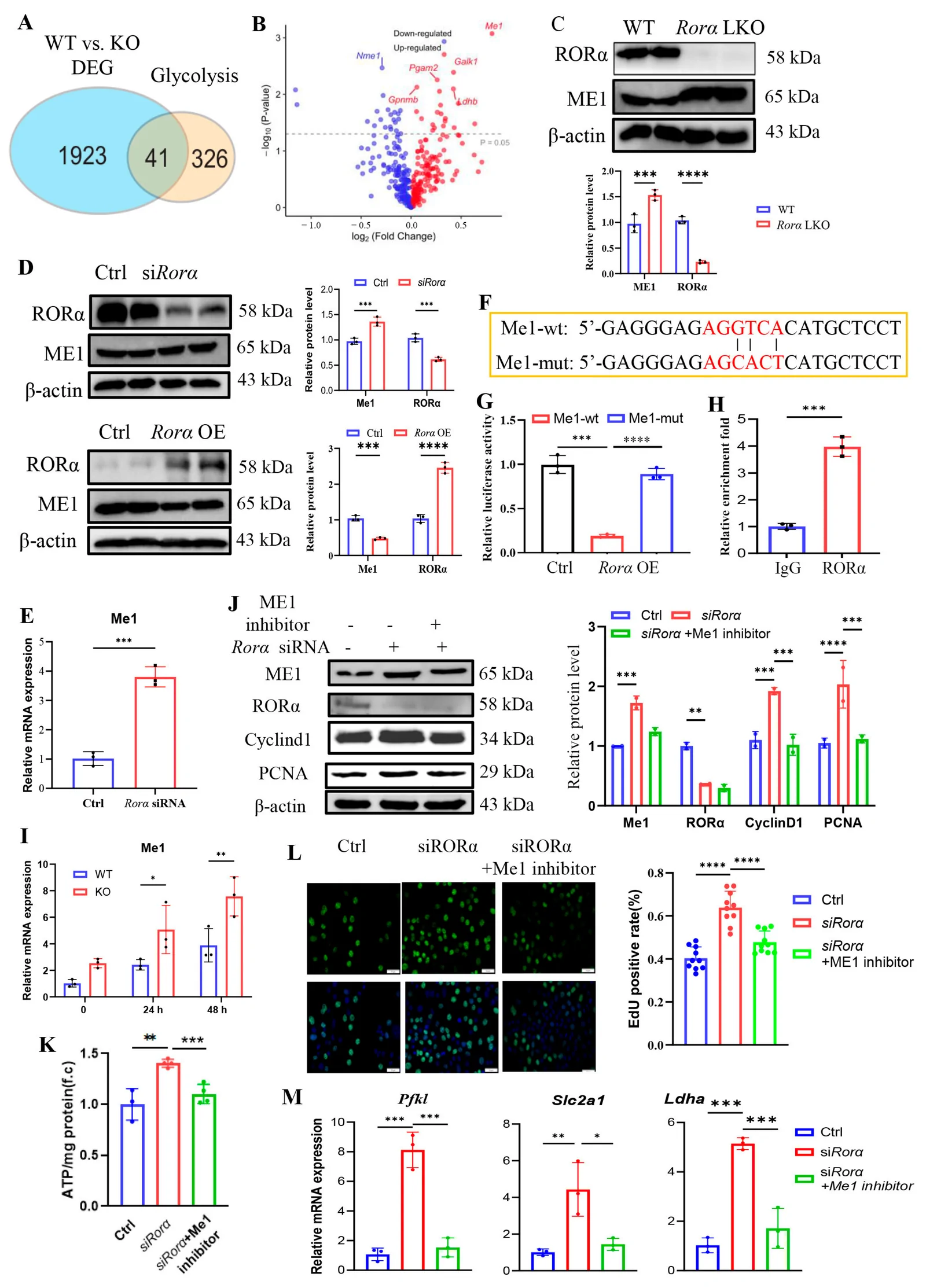
**RORα suppresses *Me1* expression to inhibit glycolysis and proliferation.** (**A**,**B**) Analysis of RNA-seq data (GSE83338) from *Rorα* WT and *Rorα*-LKO livers. (**A**) Venn diagram showing the overlap between differentially expressed genes (DEGs) and glycolysis-related genes, identifying 41 common genes. (**B**) Volcano plot highlighting the 41 glycolysis-related DEGs; *Me1* exhibited the most pronounced upregulation (Red, upregulation; Blue, downregulation). (**C**) Western blot analysis of ME1 protein levels in WT and *Rorα*-LKO livers. (**D**) Western blot of ME1 in AML12 cells following RORα knockdown or overexpression. (**E**) qPCR analysis of *Me1* mRNA levels in *siRorα* AML12 cells. (**F**) Schematic diagram of the *Me1* promoter showing the predicted RORα response element (RORE) (wt) and the mutant construct (mut). (**G**) Dual-luciferase reporter assay showing RORα-mediated repression of *Me1* promoter activity. The *Me1* promoter region was cloned into the pGL3-basic luciferase reporter vector, and the putative RORE (AGGTCA) was mutated using site-directed mutagenesis. HEK293 cells were co-transfected with the wild-type (wt) or mutant (mut) *Me1* promoter construct, together with the RORα expression plasmid or empty vector. Luciferase activities were measured using the Dual-Glo luciferase system. (**H**) Targeted CUT&Tag-qPCR analysis of RORα enrichment at the predicted RORE-containing region of the Me1 promoter. Normal IgG was used as a negative control, and exogenous spike-in DNA was used for normalization across samples. RORα enrichment is expressed relative to the IgG control. Data are presented as mean ± SD from three independent biological experiments. (**I**) qPCR analysis of hepatic *Me1* mRNA expression in *Rorα* WT and *Rorα*-LKO mice at 24 h and 48 h after PHx. (J-M) AML12 cells with RORα knockdown (siRNA) were treated with or without an ME1 inhibitor: (**J**) Western blot analysis of ME1, cyclin D1, and PCNA, (**K**) ATP levels, (**L**) EdU incorporation assay, and (**M**) mRNA expression of glycolytic genes (*Pfkl*, *Slc2a1*, *Ldha*). Data are presented as mean ± SD (n = 3–4 independent experiments or n = 3 mice per group). * *p* < 0.05, ** *p* < 0.01, *** *p* < 0.001, **** *p* < 0.0001.

To identify the downstream mediators of RORα-regulated glycolytic reprogramming, we analyzed public RNA-seq datasets GSE83338, which contains hepatic transcriptome profiles from RORα-f/f and RORα-LKO mouse [28]. A total of 1964 differentially expressed genes (DEGs) were identified between the two genotypes, of which 41 were annotated to glycolytic metabolic pathways (Figure 7A). Among these, 20 were upregulated and 21 were downregulated in RORα-LKO livers, *Me1* exhibited the most pronounced upregulation (Figure 7B), it therefore was selected as a potential downstream target of RORα. Concordantly, ME1 protein levels were significantly elevated in *Rorα*-LKO livers compared with controls (Figure 7C). In AML12 cells, RORα knockdown upregulated ME1, whereas RORα overexpression downregulated it (Figure 7D), confirming that RORα negatively regulates ME1 expression. Moreover, RORα knockdown significantly increased *Me1* mRNA abundance (Figure 7E), suggesting that RORα may regulate *Me1* at the transcriptional level.

To determine whether *Me1* is a direct transcriptional target of RORα, we used the JASPAR database to predict RORα binding sites within the *Me1* promoter and identified a putative RORα response element (RORE) (Figure 7F). Luciferase reporter assays showed that RORα overexpression suppressed the activity of a wild-type *Me1* promoter construct but failed to repress a mutant construct harboring a disrupted RORE (Figure 7G). Furthermore, targeted CUT&Tag-qPCR showed significant RORα enrichment at the predicted RORE-containing region of the Me1 promoter (Figure 7H). Collectively, these results support a model in which RORα associates with the RORE-containing region of the *Me1* promoter and negatively regulates Me1 transcription.

We next asked whether ME1 mediates the pro-proliferative effect of RORα deficiency. Hepatic *Me1* mRNA expression was markedly elevated in RORα-LKO mice at 24 h and 48 h after PHx (Figure 7I), time points that coincide with peak hepatocyte proliferation. To assess the functional contribution of ME1 to this effect, we treated siRorα AML12 cells with an ME1 inhibitor. As expected, ME1 inhibition reduced the expression of ME1, cyclin D1, and PCNA (Figure 7J), lowered intracellular ATP levels (Figure 7K), and decreased the proportion of EdU-positive cells (Figure 7L). Additionally, ME1 inhibition significantly attenuated the RORα knockdown-induced upregulation of glycolytic genes, including *Pfkl*, *Slc2a1*, and *Ldha* (Figure 7M). Together, these findings indicate that ME1 activity is required for the enhanced glycolysis and proliferation induced by RORα loss.

## 4. Discussion

In this study, we identify the nuclear receptor RORα as a critical regulator of metabolic reprogramming during liver regeneration and establish the RORα–ME1 axis as a previously unrecognized regulatory node that dictates the hepatocyte proliferation. Our findings reveal that RORα expression is dynamically regulated during the regenerative response; it increases during the early priming phase and declines as hepatocytes enter peak proliferation. Hepatocyte-specific ablation of RORα promotes hepatocyte proliferation and liver regeneration, an effect that is independent of priming-phase amplification or fatty acid oxidation but is associated with enhanced glycolysis. Mechanistically, loss of RORα relieved the transcriptional repression of *Me1*, accompanied by enhanced glycolytic flux, increased ATP availability, and accelerated hepatocyte proliferation. These findings indicate that RORα operates primarily as a cell-autonomous metabolic brake during liver regeneration.

RORα-deficient regenerating hepatocytes exhibited enhanced glycolytic features, including increased glycolysis-associated enzyme activities, altered glycolysis-related metabolites, elevated ATP levels, and increased ECAR-based glycolytic parameters. Superficially, these metabolic alterations share certain features with metabolic programs observed in many tumors [29]. However, RORα deficiency in the regenerative context accelerates recovery without increasing liver injury or inflammatory cytokine levels, in stark contrast to its tumor-promoting effects in malignancy [9,20]. Moreover, regenerative glycolysis is self-limited and resolves upon restoration of liver mass, whereas tumor glycolysis is constitutively active and drives relentless expansion [29]. These distinctions underscore that the same metabolic pathway can exert diametrically opposed biological outcomes depending on whether it is deployed in a temporally restricted regenerative program versus a chronically dysregulated neoplastic process.

Our metabolomic and functional analyses revealed reduced lipid accumulation in RORα-LKO livers without a corresponding increase in fatty acid oxidation (FAO)-related gene expression and β-hydroxybutyrate levels. This finding is particularly intriguing given the established role of transient regeneration-associated steatosis (TRAS) in supplying FAO-derived ATP during liver regeneration [23,25]. These observations suggest that RORα-deficient hepatocytes possess considerable metabolic flexibility in fueling proliferation, and that glycolysis can compensate for diminished lipid mobilization when the RORα–ME1 axis is disrupted. The preferential reliance on glycolysis may offer kinetic advantages during the early-to-mid regenerative phase, as glycolytic ATP production is more rapid than mitochondrial oxidation and generates biosynthetic intermediates (e.g., glucose-6-phosphate, 3-phosphoglycerate) required for nucleotide and lipid synthesis [30]. We acknowledge, however, that the reduced hepatic triglyceride and cholesterol levels in RORα-LKO mice may themselves be a significant contributing factor to the accelerated regenerative phenotype, rather than merely an incidental or secondary change. It is possible that reduced lipid deposition could contribute to the regenerative advantage through alleviation of lipotoxicity, altered lipid-mediated signaling, or enhanced lipid export. Our data do not exclude this possibility.

The lower basal expression of *G6pc* in RORα-LKO livers is consistent with the established role of RORα as a positive transcriptional regulator of this gene, which is known to be a direct RORα target involved in hepatic glucose production [31,32]. As G6PC catalyzes the final common step of converting glucose-6-phosphate to free glucose in both gluconeogenesis and glycogenolysis, reduced *G6pc* expression would be expected to limit the capacity of both pathways. Notably, during liver regeneration, the further suppression of *G6pc* and *Pck1* at 48 h after PHx, together with the upregulation of glycolytic genes, suggests a coordinated metabolic reprogramming that favors glucose utilization over production—a feature that may support the biosynthetic demands of proliferating hepatocytes. However, we did not directly measure hepatic glucose production or pathway-specific metabolic flux in this study. Therefore, our transcriptional data should be interpreted as suggestive evidence that RORα contributes to the basal transcriptional control of gluconeogenic/glycogenolytic genes in hepatocytes, rather than as direct proof of altered pathway activity. Direct flux analyses will be required to determine the functional consequences of this transcriptional change.

We also observed that hepatocyte-specific RORα deletion attenuates hepatic TNF-α and IL-6 levels in the PHx model, given that these cytokines are traditionally considered to originate primarily from Kupffer cells (KCs). We propose that this phenomenon reflects the dual capacity of hepatocytes to both produce and regulate these cytokines. First, hepatocytes themselves can synthesize IL-6 and TNF-α under inflammatory stimulation [33,34]. Primary hepatocytes produce TNF-α and IL-6 upon LPS challenge, and hepatocyte-derived IL-6 has been detected in the regenerating liver after PHx [35]. RORα can directly bind to the IL-6 promoter and trans-activate its transcription in certain cellular contexts [36,37], suggesting that loss of hepatocyte RORα may directly reduce hepatocyte-intrinsic IL-6 expression during the priming phase of liver regeneration. Second, and perhaps more importantly, hepatocytes actively modulate KC function through paracrine mediators. Stressed or injured hepatocytes release damage-associated molecular patterns (DAMPs) such as HMGB1, which potentially activate KCs to secrete TNF-α and IL-6 [38]. We speculate that RORα-deficient hepatocytes may exhibit altered stress responses, reduced DAMP release, or a modified secretome upon PHx, thereby diminishing KC activation and the resulting cytokine cascade. This is consistent with the emerging view that liver regeneration relies on bidirectional crosstalk between hepatocytes and non-parenchymal cells, rather than unidirectional signaling from KCs to hepatocytes [39]. Future studies using hepatocyte–KC co-culture systems and cell-type-specific transcriptomics will be needed to dissect the relative contributions of hepatocyte-autonomous vs. paracrine mechanisms in RORα-dependent cytokine regulation.

During the early post-PHx period, hepatocytes must maintain metabolic homeostasis while simultaneously preparing for cell-cycle entry [3]. The early transient upregulation of RORα after PHx may contribute to the temporal regulation of metabolic adaptation before peak hepatocyte proliferation. Its subsequent decline coincides with increased Me1 expression and enhanced glycolytic features, raising the possibility that dynamic RORα regulation contributes to the metabolic transition accompanying cell-cycle progression. This model is consistent with the observation that forced maintenance of RORα activity (via overexpression in AML12 cells) suppresses both glycolysis and proliferation, whereas its loss has the opposite effect. Future studies employing temporally controlled RORα knockdown or rescue systems will be necessary to formally test whether the early-phase expression of RORα is dispensable for, or actively protective against, regenerative failure.

The connection between RORα and ME1 offers insight into how nuclear receptors coordinate metabolic plasticity. ME1 catalyzes the oxidative decarboxylation of malate to pyruvate while reducing NADP^+^ to NADPH, thereby serving two essential functions in proliferating cells [40]. It replenishes pyruvate to sustain glycolytic flux beyond the bottleneck of pyruvate kinase, and it generates NADPH to support reductive biosynthesis and redox homeostasis. While PPARα and LXR have been extensively studied in hepatic lipid and glucose metabolism, RORα has been primarily characterized as a circadian and tumor suppressor factor [41]. Our findings expand this paradigm by supporting a transcriptional repressive role of RORα on a key anabolic enzyme, thereby bridging nuclear receptor signaling to the bioenergetic demands of tissue repair. Notably, ME1 upregulation in *Rorα*-LKO livers coincides precisely with the peak of hepatocyte proliferation (24–48 h), suggesting that the RORα–ME1 axis is not merely a static regulatory switch but a dynamically responsive module tuned to the regenerative timeline.

The transient suppression of RORα activity or pharmacological activation of the ME1–glycolysis axis could represent a novel strategy to accelerate liver regeneration in clinical scenarios where rapid restoration of functional mass is critical. These include major hepatic resection for colorectal metastases, living-donor liver transplantation, and acute liver failure, where the speed of regeneration is a major determinant of patient survival. Conversely, pharmacological enhancement of RORα signaling might be beneficial in preventing excessive or dysregulated hepatocyte proliferation, such as in the context of recurrent liver injury or preneoplastic lesions. The identification of ME1 as a druggable downstream node provides a targetable entry point for modulating regenerative metabolism without directly manipulating the nuclear receptor itself, potentially offering greater specificity and fewer off-target effects.

Several limitations of this study should be acknowledged. First, while pharmacological inhibition of ME1 attenuated the metabolic and proliferative changes associated with RORα knockdown, an independent genetic approach, such as siRNA-mediated ME1 depletion, was not performed. Therefore, the current findings support a functional contribution of ME1 but do not establish it as the exclusive mediator of the RORα-dependent phenotype. Genetic validation will be required to more definitively establish the role of ME1 in RORα-regulated metabolic reprogramming and hepatocyte proliferation. Second, we did not directly measure glycolytic or gluconeogenic flux in RORα-deficient hepatocytes or livers. Seahorse analysis was performed primarily in RORα-overexpressing cells, and direct measurements in siRORα cells or ex vivo flux assays in liver tissues were not conducted. Thus, our transcriptional and metabolomic data should be interpreted as suggestive evidence of altered pathway activity rather than as direct proof of flux changes. Finally, whether this metabolic switch confers long-term consequences for hepatocyte function or predisposes to metabolic stress upon repeated injury remains an important question for future investigation.

## 5. Conclusions

In summary, our study illuminates a novel nuclear receptor–metabolite axis in the control of tissue repair and offers a mechanistic framework for targeting metabolic reprogramming to enhance regenerative outcomes in hepatic surgery and disease.

## Data Availability

The datasets used and/or analyzed during the current study are available from the corresponding author on reasonable request.

## Supplementary Materials

The following supporting information can be downloaded at: https://www.mdpi.com/article/10.3390/genes17091029/s1, Table S1: PCR primers sequences, amplicon size, annealing temperature (AT); Table S2: qPCR primers sequences, amplicon size, annealing temperature (AT), and the specificity analysis; Table S3: Assay kits.
