# Supplementary material for: Hepatocyte-Specific RORα Deficiency Accelerates Liver Regeneration by Unleashing ME1-Driven Glycolysis
Source: Genes (Basel). 2026 Aug 28;17(9):1029. doi: 10.3390/genes17091029 (PMC13606215; doi:10.3390/genes17091029)
Supplement: Supplementary file 1 [file genes-17-01029-s001.zip › genes-4423672-supplementary.pdf]

Table S1. PCR primers sequences, amplicon size, annealing temperature (AT)

Gene	Sequence 5' to 3'	Amplicon size (bp)	Tm (°C)	AT (°C)
Rroα cDNA	F: tcgagctcaagcttcgaattcATGGAGTCAGCTCCGGCAG	1600	F: 60.6	60
	R: ttatctagagtcgcgggatccTTACCCATCGATTTGCATGGC		R: 63.1	
Me1 promoter	F: TAGGAGCTCAAAACAAGTGCTTTCC	2000	F: 60.3	60
	R: CTAGCTAGCGAGAAGCGCTGAGTCA		R: 64.2	
Me1 mutant	F: AGAGcactCATGCTCCTTGGGCTTGTTCTATAT	8000	F: 58.4	60
	R: AGAGcactCATGCTCCTTGGGCTTGTTCTATAT		R: 58.8	

F: forward primers; R: reverse primers.

Table S2. qPCR primers sequences, amplicon size, annealing temperature (AT), and the specificity analysis

Gene name‡	Sequence 5' to 3'¶	Amplicon size (bp)	Tm (°C)	AT (°C)	Melting curve
G6pc (glucose-6-phosphatase catalytic subunit)	F: CGACTCGCTATCTCCAAGTGA<br>R: GGGCGTTGTCCAAACAGAAT	173	F: 61<br>R: 61.5	60	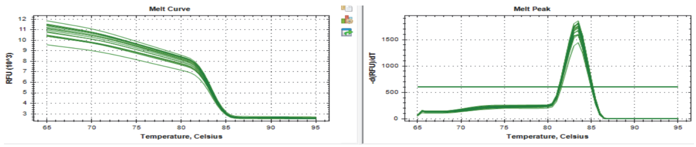
HK2 (hexokinase 2)	F: TGATCGCCTGCTTATTCACGG<br>R: AACCGCCTAGAAATCTCCAGA	112	F: 62.5<br>R: 63	60	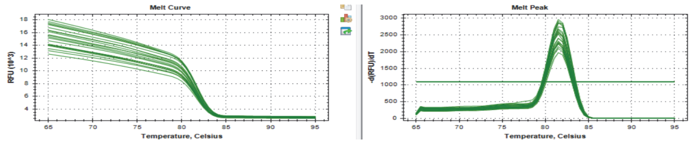
Pklr (pyruvate kinase liver and red blood cell)	F: TCAAGGCAGGGATGAACATTG<br>R: CACGGGTCTGTAGCTGAGTG	131	F: 60.3<br>R: 61.9	60	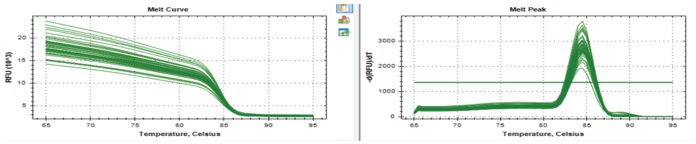
Ldha (lactate dehydrogenase A)	F: TGTCTCCAGCAAAGACTACTGT<br>R: GACTGTACTTGACAATGTTGGGA	155	F: 60.7<br>R: 60.2	60	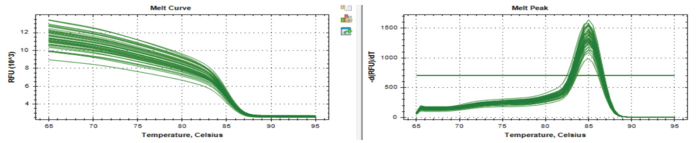
Pck1(phosphoenolpyruvate carboxykinase 1, cytosolic)	F: CTGCATAACGGTCTGGACTTC<br>R: CAGCAACTGCCCGTACTCC	159	F: 60.2<br>R: 62.7	60	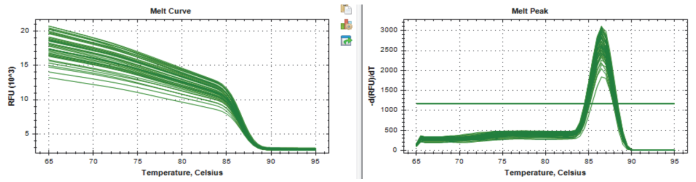

Pfkl (phosphofructokinase, liver, B-type)	F: GGAGGCGAGAACATCAAGCC<br>R: CGGCCTTCCCTCGTAGTGA	113	F: 62.9<br>R: 63	60	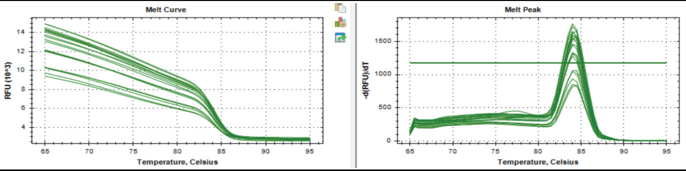
Slc2a1 (solute carrier family 2, member 1)	F: CAGTTCGGCTATAACACTGGTG<br>R: GCCCCCGACAGAGAAGATG	156	F: 60.1<br>R: 61.8	60	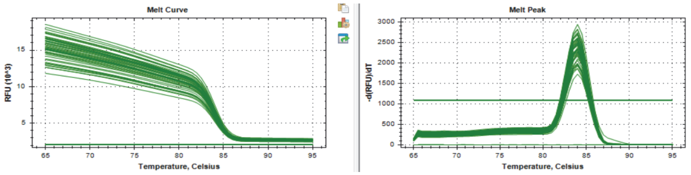
Slc2a2 (solute carrier family 2, member 2	F: TCAGAAGACAAGATCACCGGA<br>R: GCTGGTGTGACTGTAAGTGGG	215	F: 60.2<br>R: 62.6	60	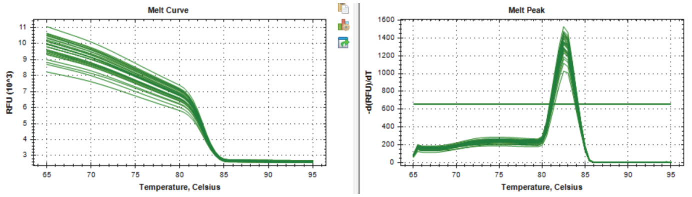
Slc16a3 (solute carrier family 16, member 3)	F: TCACGGGTTTCTCCTACGC<br>R: GCCAAAGCGGTTCACACAC	167	F: 61.4<br>R: 62.2	60	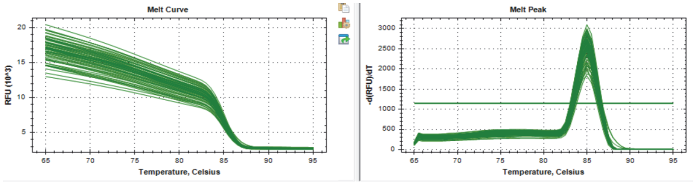
Il6 (interleukin 6)	F: AGTTGCCTTCTTGGGACTGA<br>R: TCCACGATTTCCCAGAGAAC	159	F: 58.8<br>R: 57.2	60	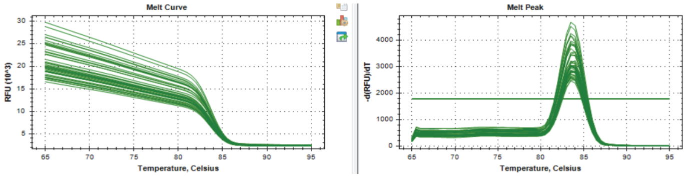
RORα (RAR-related orphan receptor alpha)	GTGGAGACAAATCGTCAGGAAT<br>TGGTCCGATCAATCAAACAGTTC	135	F: 60<br>R: 60.8	60	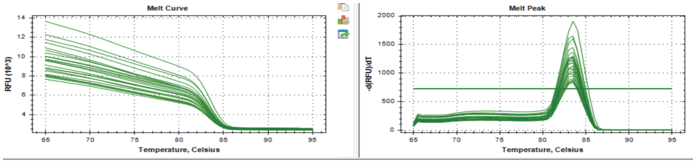

Fgf21 (fibroblast growth factor 21)	CTGCTGGGGGTCTACCAAG<br>CTGCGCCTACCACTGTTCC	154	F:61.4<br>R:62.7	60	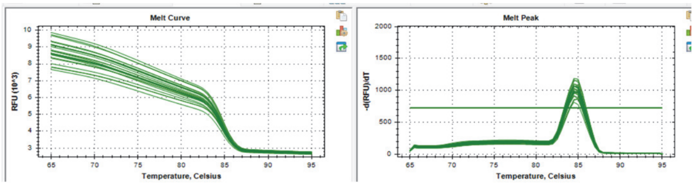
Tkt (transketolase)	F: ATGGAAGGTTACCATAAGCCAGA<br>R: TGCAGCATGATGTGGGGTG	127	F: 60.8<br>R: 62.7	60	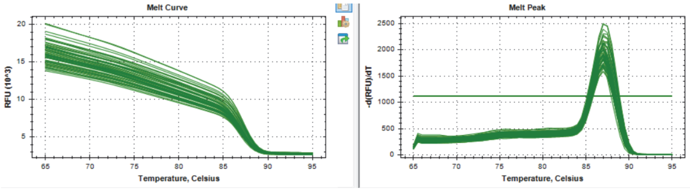
TGFα (transforming growth factor alpha)	F: CACTCTGGGTACGTGGGTG<br>R: CACAGGTGATAATGAGGACAGC	136	F: 61.7<br>R: 60.4	60	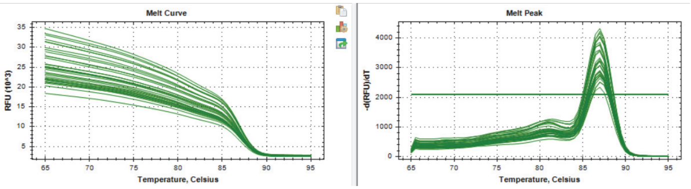
Apoa1 (apolipoprotein A-I)	F: GGCACGTATGGCAGCAAGAT<br>R: CCAAGGAGGAGGATTCAAACTG	129	F: 62.9<br>R: 60.3	60	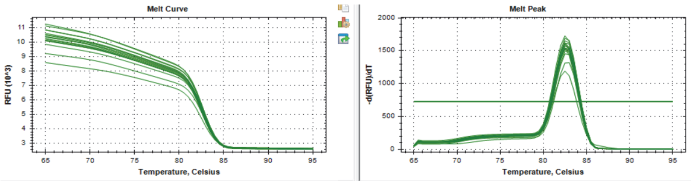
Hgf (hepatocyte growth factor)	F: ATGTGGGGGACCAAACTTCTG<br>R: GGATGGCGACATGAAGCAG	79	F: 62<br>R: 60.9	60	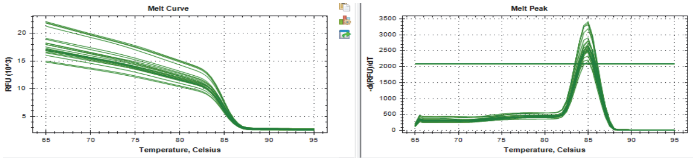
Me1 (malic enzyme 1, NADP(+)-dependent, cytosolic)	F: GTCGTGCATCTCTCACAGAAG<br>R: TGAGGGCAGTTGGTTTTATCTTT	102	F: 60.4<br>R: 60.1	60	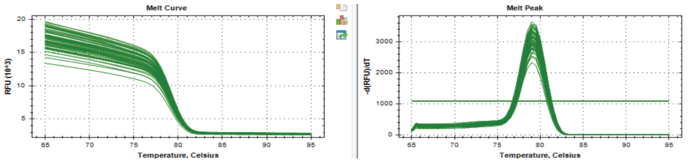

Tnfα (tumor necrosis factor a)	F: ACTGAACTTCGGGGTGATCG<br>R: GGTGGTTTGTGAGTGTGAGG	101	F: 59.7<br>R: 58.6	60	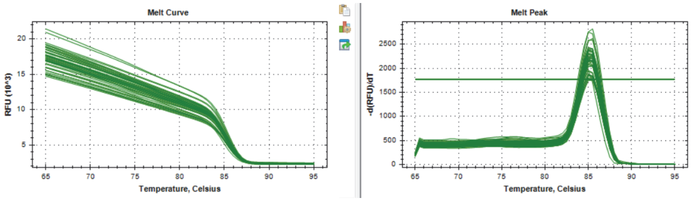
18s (18S ribosomal RNA)	F: CGCCGCTAGAGGTGCAATTC<br>R: CCAGTCGGCATCGTTTATGG	150	F: 61.7<br>R: 59	60	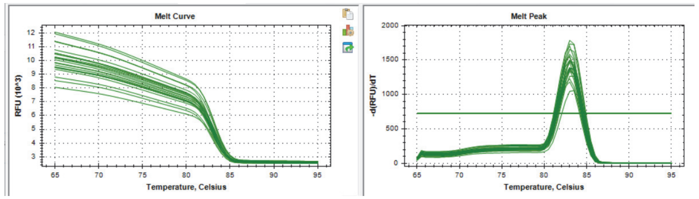
Cut&Tag					
Me1 promoter RORE-containing region	F: TCCTTGGGCTGCTCACC<br>R: TTTAGAGATGTTGCTTGTCCATTGC	206	F: 63.1<br>R: 63.9	60	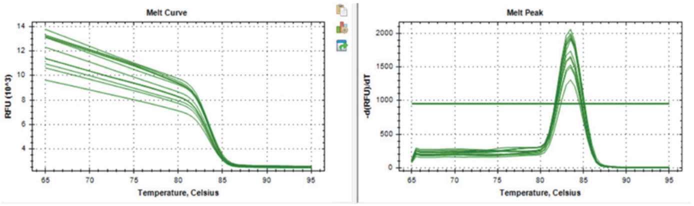
DNA spike-in	F: GTACATCGCAAAGTCTCCGC<br>R: ATATGGGCGCAGATAGCACAT	122	F: 58.8<br>R: 58.8	60	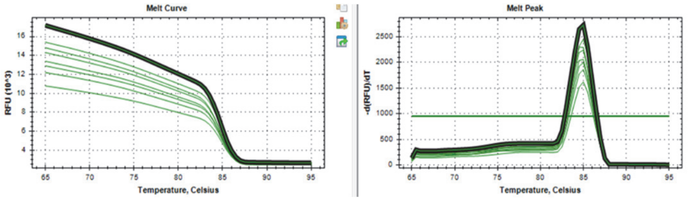

‡The untargeted metabolomics analysis identified glycolysis as the most significantly altered pathway in RORα-deficient livers following PHx. Therefore, a panel of genes representing multiple key steps in glucose utilization and glycolytic metabolism were selected for expression analysis. Specifically, *Slc2a1* (GLUT1) and *Slc2a2* (GLUT2) were examined to assess glucose transport; *Hk2*, *Pfkl*, and *Pklr* represent major enzymatic steps in the glycolytic pathway; *Ldha* reflects the conversion of pyruvate to lactate; and *Slc16a3* (MCT4) is involved in lactate export. *G6pd* and *Tkt* were included to assess the oxidative and non-oxidative branches of the pentose phosphate pathway, respectively, as this pathway is closely linked to glucose utilization and provides reducing equivalents and biosynthetic intermediates during cell proliferation. ¶F: forward primers; R: reverse primers.

Table S3. Assay kits

Regents or kits	Company	Catalog No.
Alanine transaminase (ALT)	Beyotime Institute of Biotechnology (Shanghai, China)	P2711
Aspartate transaminase (AST)		P2715
Mouse TNFα ELISA kit	EIAAB Science (Wuhan, China)	E0087m
Mouse IL6 ELISA kit		E0079m
Phosphofructokinase (PFK)	Solarbio Life Sciences (Beijing, China)	BC0535
pyruvate kinase (PK)		BC0540
hexokinase (HK)		BC0745
